# Enterprise service user intent prediction based on fast K-means++ fusion algorithm

**DOI:** 10.1371/journal.pone.0334478

**Published:** 2026-04-27

**Authors:** Yuanyuan Han, Juanjuan Zhai, Ping Li

**Affiliations:** Institute of Digital Economy and Smart Management, Huanghe Jiaotong University, Jiaozuo, China; Kaplan Business School of Australia, AUSTRALIA

## Abstract

To address the low efficiency of feature mining and limited prediction accuracy in enterprise service user intent prediction, a research proposes an enterprise service user intention prediction model that integrates heuristic variants inspired by Kmeans++and Stacking ensemble learning. The model improves traditional K-means++ clustering through adaptive weighted grid information entropy optimization, solving the problems of slow convergence and uneven weight distribution in large-scale data. It also builds a weighted ensemble learner using base classifiers such as random forest to enhance intent prediction performance after multidimensional feature fusion. The experimental results show that the optimized Fast K-means++clustering algorithm achieved a contour coefficient of 0.92, a Calinski Harabasz index of 2500, and a Davies Bouldin index of 0.12 on dense point datasets, with significantly better clustering quality than the comparative algorithms. In the testing of the FK Stacking prediction model in real e-commerce scenarios, the accuracy, recall, and F1 score all exceeded 0.97, and the error rate remained stable below 2.1% in medium and long-term time series predictions; After iterative optimization, the model’s memory usage was reduced by 50% and response time was shortened by 82.5%. The results show that the proposed model offers lightweight and high-accuracy advantages in enterprise service user data analysis and intent prediction. It can help enterprises optimize resource allocation and improve service response speed.

## 1. Introduction

With the wave of digital transformation sweeping across the globe, the behavior patterns of enterprises serving users are becoming increasingly complex and diverse. In this context, accurately predicting user intentions has become a key link in enhancing customer lifetime value and optimizing service experience [1]. When user intent undergoes structural changes, accurate prediction can help enterprises avoid risks and explore new opportunities [2]. However, achieving accurate intent prediction faces many challenges. Traditional methods, such as linear regression, struggle to capture high-dimensional dynamic user behavior. Single clustering algorithms also suffer from low efficiency and randomness in initial center selection when handling large-scale data [3]. K-means++ improves clustering quality by optimizing initial center selection, which enhances processing speed for large-scale datasets. However, it still faces high distance computation complexity in scenarios with large weight differences [4]. Stacking ensemble learning combines the strengths of multiple models, but existing studies lack dynamic weight optimization for base classifier outputs, which limits prediction accuracy [5]. To solve these problems, this paper introduces an improved K-means++ method optimized by adaptive weighted grid information entropy. It adjusts grid density dynamically and quantifies it through entropy to enhance clustering efficiency. Based on the error rates of base classifiers, the model dynamically assigns weights to reduce overfitting in the Stacking ensemble learner. Combining these two approaches, this model aims to efficiently and accurately support user group profiling and dynamic intent prediction in enterprise services. The proposed method is expected to offer a new technical path for real-time user demand response and refined enterprise operations.

## 2. Related works

In recent years, with the rapid development of computer technology, the K-means++ algorithm has shown several advantages compared with other algorithms, such as high efficiency in processing large-scale data and improved quality of initial centers. It has played an important role in various fields, and many scholars both in China and abroad have studied it. To determine the optimal distance computation in the K-means algorithm, Buaton et al. raised the K-means++ algorithm, which randomly selects the first cluster center from the data and then performs seven rounds of distance calculations based on numerical measurement types [6]. To fully consider potential changes among samples, Liu put forward a K-means clustering framework that parameterizes the domain mask matrix with a set of hyperparameters. The framework learns domain mask coefficients through clustering tasks and solves them using a gradient-based method [7]. For traffic flow prediction, which can greatly relieve congestion pressure, Sun et al. developed a combined method of K-means and gated recurrent unit. This method clusters historical traffic data to build different traffic libraries and then uses a classification algorithm to identify the most similar historical day for prediction [8]. The Fast algorithm significantly enhanced model performance by its efficiency and lightweight nature in preferred feature detection. To achieve responsiveness in Byzantine fault tolerance, Jalalzai et al. integrated Fast into their method. The algorithm decomposed n data samples into single data units and executed different units in the same batch, achieving optimal run time with minimal memory usage [9]. In the field of educational outcome evaluation, Hendrastuty et al. used silhouette scores to evaluate clustering results and identify optimal cluster structures. Their experiments on actual student assessment data demonstrated the effectiveness of silhouette scores in clustering analysis and data interpretation [10].

Customer intent often shapes the strategic direction of enterprises, and it is influenced by both internal and external factors. To predict customer intent, researchers have explored clustering, robotic learning models, and knowledge graphs from multiple perspectives. To address the challenge of predicting human motion behavior in robotics, Baruah et al. proposed a proxy model driven by sensory prediction errors. The model minimized both classification and generative errors to learn the sequence of body positions [11]. For pedestrian recognition in autonomous driving systems, Yang et al. introduced a stack of recurrent neural networks. Their model integrated different sensing strategies and established a spatiotemporal feature-based framework [12]. Meharie et al. developed an ensemble model combining linear regression, support vector machine, and artificial neural network for highway construction cost prediction. The results showed that this model achieved lower prediction errors than individual models [13]. To address the complexity of network management and configuration, Leivadeas et al. proposed an intent-aware network. This network separated instance-related and category-related features during batch normalization, reducing internal covariate shift and improving generalization [14]. To predict the impact of chatbot perception on user intent, Song et al. put forward the uncanny valley hypothesis. Their model enhanced visual realism and animation to shape chatbot image and forecast potential negative effects on user trust and intent [15]. Zhang et al. proposed a novel deep adaptive evolutionary ensemble (DAEE) model based on deep forest algorithm and further integrated evolutionary ensemble learning method. This model introduces model diversity in the cascading layers, enabling it to adaptively adjust its structure to adapt to complex and constantly changing purchasing behavior patterns. The results indicate that the research model not only has higher robustness, but also has an AUC value increase of 5.02% compared to the baseline model [16]. Da proposed a prediction method for e-commerce consumers’ purchase intention in online marketing to improve the accuracy of prediction results. Firstly, collect consumer related data, standardize the data, and handle outliers. Secondly, use logistic regression algorithm to extract and select consumer characteristics related to purchase intention, revealing consumers’ purchasing preferences. Finally, based on the feature extraction results, the random forest algorithm is used to predict purchase intention. The research results show that the root mean square error of the proposed method is relatively low, with the highest standardized information value reaching 0.89, indicating that it can accurately reflect consumers’ purchase intention [17]. Lian et al. designed a competitive framework for predicting pedestrian crossing intentions using only video sequences obtained from RGB cameras installed on vehicles in natural traffic scenes. The simulation results show that the accuracy of the model reaches 89.68%, and its performance is better than related works, with an accuracy of 89.68% [18].

In summary, although some progress has been made in enterprise service user intent prediction, current technologies still face challenges such as low efficiency in user feature mining and insufficient accuracy after multidimensional feature fusion. The K-means++ algorithm can significantly reduce invalid iterations and improve speed and accuracy in processing large-scale data. The weighted ensemble learner built by Stacking ensemble learning can greatly strengthen the prediction capability after feature fusion. Therefore, this paper proposes a prediction model for enterprise service user intent by integrating K-means++ with Stacking ensemble learning. This model is expected to improve both the efficiency and accuracy of user intent prediction and provide technical and data support for building user interaction data-driven prediction models.

## 3. Construction of enterprise service user intent prediction model

### 3.1. K-means++ clustering optimization based on adaptive weighted grid information entropy

**Fig 1:**
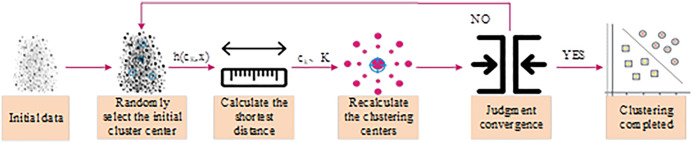
Algorithmic process of K-means++ clustering.

In enterprise service user intent data analysis, K-means often faces problems such as excessive iterations and high computational complexity. These problems are especially serious when dealing with massive volumes of user intent data, where the algorithm is likely to experience a bottleneck in convergence speed. K-means++ introduces an initial center selection strategy based on data distribution, which significantly reduces invalid iterations and improves the speed and accuracy of large-scale data processing [19]. The algorithmic process of K-means++ clustering is shown in Fig 1.

As shown in Fig 1, the standard K-means++clustering first randomly selects the first cluster center from the input data. Subsequently, calculate the shortest distance from the remaining data points to the selected center, and iteratively select a new center based on distance weighted probability. Obtain the optimal clustering center through continuous iteration [20]. Among them, the initial clustering center expression is shown in equation (1).


$$c_{K}=\left\{ \begin{array}{c} @c@h(s,x{)}_{min}\\ k*h(s,x{)}_{max} \end{array},k\in \left[0.5\sim \;1\right]\right.$$ (1)

In Equation 1, c represents the initial cluster center, x denotes the sample, h(s,x) is the Euclidean distance between s and the vector, and k is a correction factor. After confirming the initial cluster center, the calculation of the composite coefficient of variation is shown in Equation 2.


$$c_{v}=\frac{\mu_{outer}*\sigma_{inner}}{\sigma_{outer}*\mu_{inner}},\sigma ,\mu \in \left[0,1\right]$$ (2)

In Equation (2), $c_{v}$ represents the composite coefficient of variation, $\mu_{outer}$ and $\sigma_{outer}$ represent the mean and variance of the partitioned samples, while $\mu_{inner}$ and $\sigma_{inner}$ represent the mean and variance of the retained samples. The change rate of the initial cluster center is given in Equation (3).


$$h_{Rj}=\left|\frac{h_{i}-h_{j}}{h_{j}}\right|,h\left(\Delta c\right)<1,j=i+1$$ (3)

**Fig 2:**
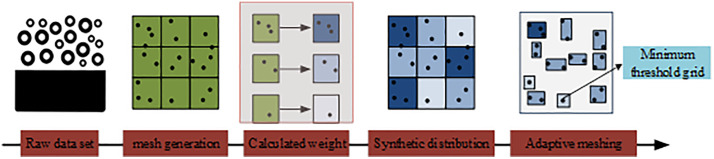
Principle of data partitioning using adaptive weighted grid information entropy.

In Equation (3), $h_{Rj}$ represents the distance change rate of the j -th cluster center, and Δc represents the distance difference between two centers. Although K-means++ improves clustering quality by optimizing the selection of initial centers, it still faces problems such as high distance computation complexity and slow convergence when processing enterprise user intent data with significant weight differences. Adaptive weighted grid information entropy maps user intent data into a predefined grid space using a dynamic grid partitioning strategy. It refines grids in dense areas and coarsens them in sparse regions, highlighting core clustering areas and significantly improving algorithm performance in large-scale data environments [21]. The working principle of adaptive weighted grid information entropy is shown in Fig 2.

As shown in Fig 2, the process of handling data through weighted grid information entropy includes uniform grid partitioning, grid density calculation, individual grid weight allocation, and adaptive adjustment of grid size and density. Weighted grid information entropy better reflects the occurrence probability of events in a system. Many clustering algorithms can assign weights based on grids to efficiently identify core objects and neighborhoods, thereby enhancing clustering and data mining efficiency. In practical applications, clustering performance often depends on grid partitioning parameters. The calculation of weighted grid partitioning is given in Equation (4).


WG=(G,N(g),W) (4)

In Equation (4), g represents a unit grid, and N(g) denotes the set of associated cells. The information entropy of a unit grid is calculated using Equation (5).


H(X)=−∑p(x)×lbp(x) (5)

In Equation (5), X represents a discrete variable, and p(x) is the probability of the variable occurring in a system event. When data points within a grid are distributed too densely or too sparsely, the grid needs to be expanded or contracted. The partitioning threshold determines the density of data distribution in the current grid and reflects the overall data distribution within it. The calculation of the partitioning threshold is shown in Equation (6).


$$\varphi =\mu \times min\left(\frac{\text{1}}{n}\sum_{\begin{array}{c} i=1\\ i\neq j \end{array}}^{n}\|p_{i}-p_{j}\|\right)$$ (6)

In Equation (6), μ is the number of data points in the smallest unit grid, and $p_{i}$ and $p_{j}$ represent individual data points at different positions. The function used to compute grid size based on the threshold is given in Equation (7).


$$h\left(x\right)=h_{\text{0}}\times (1+\alpha \|\nabla \mu h\left(x\right)\|{)}^{-\beta }$$ (7)

**Fig 3:**
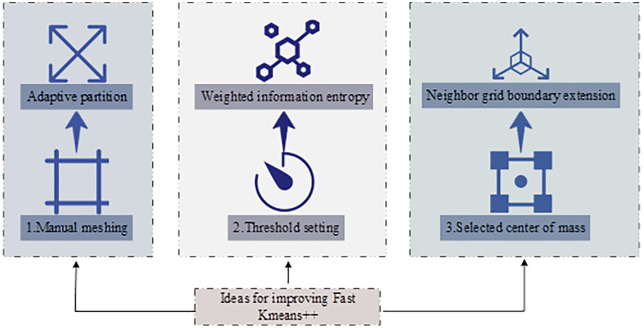
Key points for improving clustering speed in Fast K-means ++ data analysis (Icon source from: https://www.aigei.com/).

In Equation (7), h(⬝) is the grid size function used to control local grid density, and $h_{\text{0}}$ is the initial grid size determined by dimension. By rapidly locating high-density regions using a weighting factor and quantifying the information entropy, the algorithm reduces invalid distance computations. This process helps K-means++ form a more efficient candidate set of initial centers during preprocessing and significantly boosts performance in large-scale data environments. Therefore, this study optimizes K-means++ with weighted grid information entropy and proposes a fast clustering algorithm named Fast K-means++. This algorithm is based on heuristic principles and empirical data, rather than a theoretically guaranteed variant of K-means++. The key points for performance improvement in data analysis clustering using Fast K-means++ are shown in Fig 3.

As shown in Fig 3, Fast K-means++ mainly optimizes three aspects: grid partitioning, minimum density threshold, and center selection in dense datasets. Adaptive grid partitioning improves clustering density in unit grids. The specific process is as follows. Firstly, the user intent data is adaptively meshed, and the mesh granularity is dynamically adjusted according to the data distribution density, that is, the mesh is refined in dense areas and coarsened in sparse areas. Next, calculate the data density of each grid cell and calculate the weighted information entropy accordingly. Use the preset minimum density threshold to screen high-density grid cells and form a high-quality initial cluster center candidate set. The construction of the candidate set aims to identify regions with high clustering potential. The specific process is as follows. Firstly, the data space is divided using an adaptive grid strategy, where the granularity of the grid is dynamically determined by the local density of the data points: regions with higher concentration of data points are divided into smaller and more detailed cells to preserve structural details, while sparse regions are assigned larger and coarser cells to reduce computational overhead. After establishing the grid structure, the density of each grid cell is calculated based on the number of data points contained therein, and the weighted information entropy of each cell is calculated using this density information, which reflects the uniformity of data distribution within the cell and serves as an indicator of its suitability as an initialization center. Then, a minimum density threshold is applied to filter the grid cells; only those cells with a density reaching or exceeding this threshold are retained as high-density candidate regions. Finally, all data points within the retained high-density grid cells are extracted and aggregated to form the candidate set used for initial center selection. Points within cells below the density threshold are excluded during the initialization stage, but they remain part of the dataset and will be assigned to different clusters in the subsequent iterative optimization stage. This candidate set construction mechanism ensures that the initialization process can concentrate computing resources on the regions most likely to contain meaningful clustering centers, thereby improving efficiency and clustering quality. Then, an optimized initial center selection process is performed within the candidate set: after randomly selecting the first initial center, a probability distribution is constructed based on the shortest distance between the data points and the existing centers, and the remaining centers are selected sequentially. This restricts the high computational complexity of distance operations to a limited high-density candidate set, significantly improving efficiency. After determining K initial centers, the algorithm enters the restricted K-means iteration stage, sequentially assigning data point clusters and updating cluster centers until the rate of change in cluster centers stabilizes or reaches the maximum number of iterations, and finally outputs the clustering results.

The minimum density threshold determined by weighted information entropy eliminates grids that do not meet the partitioning criteria. Dense datasets expanded by weighted grids simplify the center extraction process. These improvements collectively reduce computational complexity and enhance the accuracy and efficiency of Fast K-means++ [22]. The density calculation for grid units and the minimum threshold is given in Equation (8).


$$H\left(M\right)=-\sum_{P}\left(m\right)Ibp\left(m\right)$$ (8)

In Equation (8), m represents the density of data in a grid unit after grid-based processing, and p(m) is the probability of m occurring. The calculation of weighted grid information entropy in Fast K-means++ is shown in Equation (9).


$$H^{\prime }(M)=-\sum_{l=1}^{m}P(den(U)=a)\times IbP(den(U)=a)$$ (9)

In Equation (9), count(a) represents a grid with density a, and count(l) is the total number of divided grids. The equation for removing grid units that do not meet the minimum threshold is shown in Equation (10).


$${H^{\prime }}^{\prime }(M)=-\sum_{l=1}^{l}P(den(U)=a)\times IbP(den(U)=a)\times \frac{\sum_{l=1}^{n}\frac{a_{l}}{n}}{a}$$ (10)

In Equation (10), ${H^{\prime }}^{\prime }\left(M\right)$ represents the density threshold for a grid unit and serves as a reference for filtering out non-compliant data units.

### 3.2. Enterprise service user intent prediction model combining stacking and Fast K-means++

**Fig 4:**
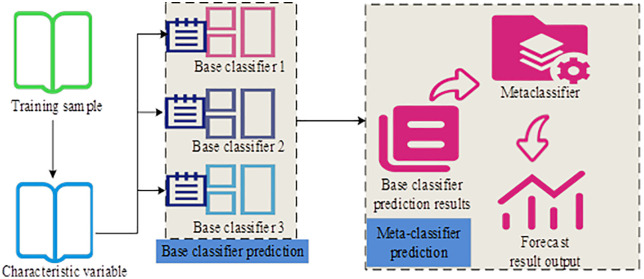
Stacking process of multiple classifiers (Icon source from: https://www.aigei.com/).

Although Fast K-means++ improves computational efficiency, it still struggles to capture the multidimensional features and latent associations of user intent when used as a single clustering algorithm. Stacking ensemble learning combines the prediction results of multiple base models to extract deeper data patterns, offering enterprises a more efficient and robust solution for accurate user intent prediction. The process of data stacking through multiple classifiers in the Stacking algorithm is shown in Fig 4.

In Fig 4, training samples are first processed by multiple base classifiers, each producing a prediction result. These outputs are then used as the input features for a meta-classifier, which generates the final prediction result. The performance of Stacking depends on the choice of base classifiers [23]. This study adopts extreme Gradient Boosting (XGBoost), Random Forest (RF), and Gradient Boosting Decision Tree (GBDT) as base learners, and Logistic Regression Model (LRM) as the meta-learner [24–26]. The formulation of GBDT is shown in Equation (11) [27].


$$F_{a}\left(x\right)=\sum_{i=1}^{m}T(x;\theta_{a})$$ (11)

In Equation (11), a represents the number of prediction rounds, and $T(x;\theta_{a})$ is the number of weak classifiers. GBDT improves accuracy by using the residuals from each weak classifier. XGBoost follows a similar principle to GBDT but reduces the loss value during node splitting. The loss reduction function is shown in Equation (12).


$$max\frac{\text{1}}{\text{2}}\frac{{G_{L}}^{\text{2}}}{H_{L}+\lambda }+\frac{\text{1}}{\text{2}}\frac{{G_{R}}^{\text{2}}}{H_{R}+\lambda }-\frac{\text{1}}{\text{2}}\frac{(G_{L}+G_{R}{)}^{\text{2}}}{G_{L}+H_{R}+\lambda }-\lambda$$ (12)

**Fig 5:**
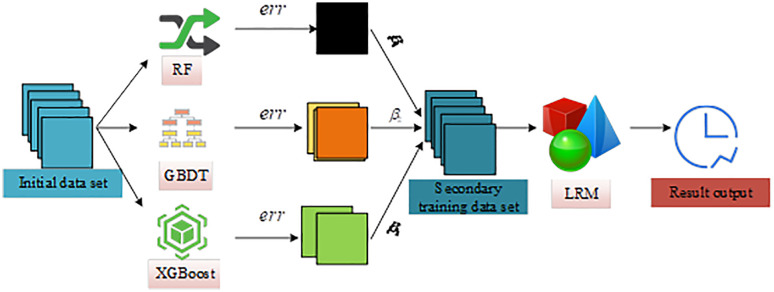
Structure of weighted ensemble learner in Stacking (Icon source from: https://www.aigei.com/).

In Equation (12), L is the loss function, and λ is the regularization coefficient. Directly using the outputs of base classifiers to train the meta-learner may lead to overfitting. Assigning different weights to the base classifiers based on their performance helps prevent overfitting. Therefore, before the second training stage of Stacking, this study assigns weights to the outputs of base classifiers according to their error rates. The structure of the weighted ensemble learner combining XGBoost, RF, GBDT, and LRM is shown in Fig 5.

As shown in Fig 5, the model calculates a weight coefficient for each base classifier after generating prediction results. The weight is determined by the error distribution of each classifier on the initial samples. This process helps improve the overall prediction accuracy of LRM. The error rate of base classifiers is calculated using Equation (13).


$$err=\frac{\text{1}}{c}\left(\sum \frac{n}{N}\right)$$ (13)

In Equation (13), err denotes the error rate, while n and c represent the number of misclassified samples and the number of cross-validation folds, respectively. During the second training stage, the weight coefficient that influences the meta-classifier is computed as shown in Equation (14).


$$\left\{ \begin{array}{c} @c@\beta =\frac{1-err}{err}\\ g=\frac{\beta }{\sum \beta } \end{array}\right.$$ (14)

**Fig 6:**
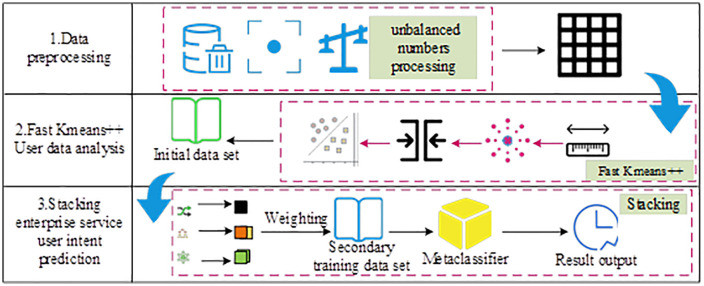
Operation process of FK-Stacking model (Icon source from: https://www.aigei.com/).

In Equation (14), β and g represent the learning weight based on the error rate and the corresponding training weight proportion. This study integrates Fast K-means++ with the weighted ensemble learner of Stacking to construct an enterprise service user intent prediction model, named FK-Stacking. The model uses Fast K-means++ to cluster users into subgroups such as high-value clusters and potential churn clusters. For each cluster, a separate Stacking model is built. The internal characteristics of each cluster are used to optimize prediction accuracy [28–29]. The operation process of the FK-Stacking model is shown in Fig 6.

As shown in Fig 6, the model performs data preprocessing to reduce the impact of missing values, outliers, and imbalanced data. It uses adaptive weighted grid information entropy to enhance the representation of enterprise service user intent and employs Fast K-means++ clustering to extract clear user feature samples. The clustering results are used to train the base classifiers, whose weighted predictions serve as the training input for the meta-learner, producing a comprehensive prediction output. To address class imbalance, the model applies the k-nearest neighbor method, which generates new data based on the Euclidean distance of minority class samples. The corresponding calculation is shown in Equation (15) [30].


$$dist\left(B,C\right)=\sqrt{\sum_{m}^{n}(b_{i}-c_{i}{)}^{\text{2}}}$$ (15)

In Equation (15), b represents minority class samples, and c represents the other samples. The construction of new samples from imbalanced data is shown in Equation (16).


$$\overset{―}{b}=b+rand\left(0,1\right)*(\tilde{b}-b)$$ (16)

In Equation (16), $\tilde{b}$ is a neighboring sample of b, and $\overset{―}{b}$ is the newly generated sample. The proposed integrated prediction model optimizes performance across data selection, feature classification, and result training. As a result, it improves the model’s ability to recognize features and enhances prediction accuracy.

## 4. Comprehensive evaluation of model performance

### 4.1. Performance testing of fast K-means++ clustering algorithm

To evaluate the performance of the Fast K-means++ algorithm in data analysis and clustering, this study conducted simulation experiments using both synthetic datasets generated by MATLAB functions and the Iris dataset from the UCI repository. The Iris dataset contains 150 samples, each with 4 features (sepal length, sepal width, petal length, petal width), for a total of 3 categories. In addition to the standard dataset, the study also included a spiral dataset (sample size: 1000, dimension: 2), a clustering dataset (sample size: 800, dimension: 2), and a dense point letter dataset (sample size: 1200, dimension: 2) to verify the mining ability and robustness of the algorithm under different data structures. The clustering algorithms used for comparison were Gradient Descent Spotted Hyena Optimizer K-means (GD-SHO-K-means), Simulated Annealing Seagull Optimization Algorithm K-means (SA-SOA-K-means), and Sand Cat Swarm Optimization K-means (SCSO-K-means). The experimental environment included an Intel i7 processor, NVIDIA A100 GPU, 64GB RAM, SSD high-speed storage, and Windows 10 operating system. The Silhouette Score (SS) measured how well each data point matched its assigned cluster, with higher values indicating better results. The Calinski-Harabasz (CH) index evaluated clustering compactness and separation based on the ratio of inter-cluster and intra-cluster variance. The Davies-Bouldin (DB) index assessed cluster similarity, where lower values indicated better performance. The comparison of these metrics across algorithms and datasets is shown in Table 1.

**Table 1:** Comparison of clustering performance on different data types.

Performance index	Data type	Fast Kmeans++	GD-SHO-Kmeans	SA-SOA-Kmeans	SCSO-Kmeans
SS	Dense point	0.92	0.78	0.73	0.85
	Clustered	0.81	0.69	0.65	0.74
	Spiral shape	−0.05	−0.12	−0.21	−0.08
CH	Dense point	2500	1800	1500	2200
	Clustered	1800	1200	1000	1500
	Spiral shape	300	150	100	200
DB	Dense point	0.12	0.18	0.23	0.15
	Clustered	0.20	0.30	0.35	0.25
	Spiral shape	0.90	1.10	1.30	1.00

**Fig 7:**
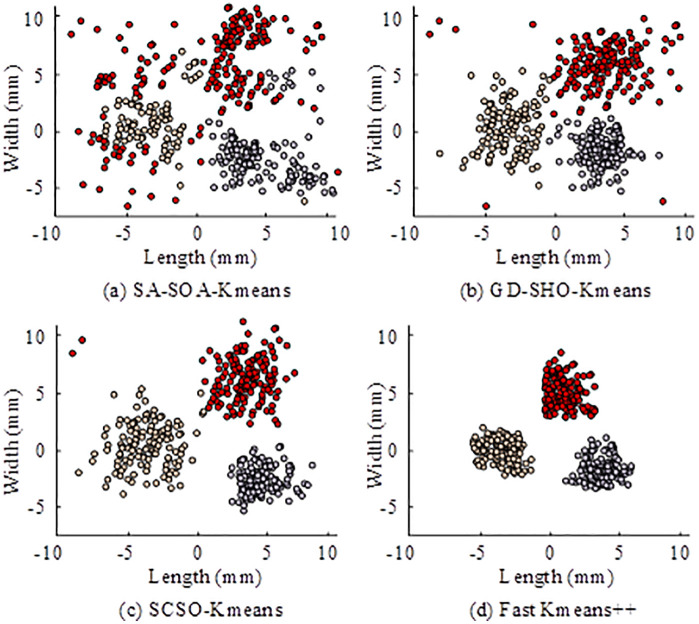
Clustering result visualization of different algorithms.

As shown in Table 1, for dense-point datasets, Fast K-means++ achieved an SS of 0.92, CH of 2500, and DB of 0.12, indicating optimal compactness and separation. On clustered data, SA-SOA-K-means reached an SS of 0.65 and CH of 1500, both lower than Fast K-means++, and Fast K-means++ showed the lowest cluster similarity, significantly outperforming the alternatives. For spiral data, all algorithms had negative SS values, but Fast K-means++ showed a more dispersed centroid distribution and still outperformed the others. These results indicated that the proposed algorithm consistently performed better on convex datasets and maintained a clear advantage even in more complex, non-convex scenarios. A visualization of clustering results is shown in Fig 7.

**Fig 8:**
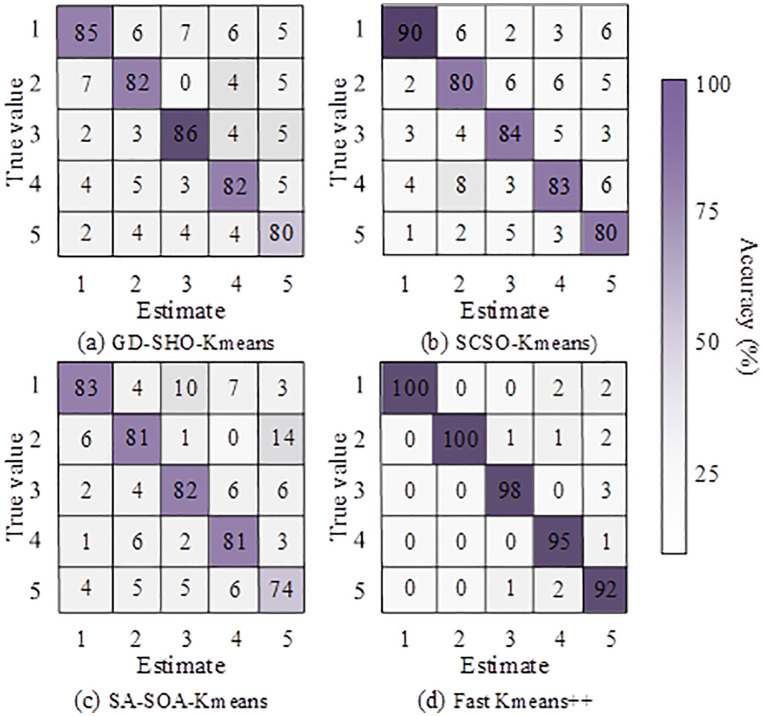
Confusion matrix comparison of five-feature recognition by different algorithms.

In Fig 7(a), the red-brown clusters of SA-SOA-K-means overlapped heavily, and the gray clusters appeared loose with unclear boundaries, showing flaws in local optimization. In Fig 7(b), GD-SHO-K-means left outlier points in the red cluster, while brown and gray clusters still overlapped, indicating convergence bottlenecks during gradient optimization. In Fig 7(c), SCSO-K-means reduced red-brown overlap and improved gray cluster compactness, showing better performance but still limited refinement. In Fig 7(d), the three clusters were completely separated with clear boundaries, effectively avoiding overlap and producing the best result. These findings confirmed that Fast K-means++ significantly outperformed other algorithms in terms of separation and compactness. The study further analyzed the confusion matrices of five-feature recognition using the selected algorithms, as shown in Fig 8.

In Fig 8(a), GD-SHO-K-means had an average diagonal accuracy of 83%, with only 80% accuracy in identifying the fifth feature, indicating moderate classification stability. In Fig 8(b), SCSO-K-means achieved diagonal accuracy ranging from 80% to 90%, improving over the previous algorithm. In Fig 8(c), SA-SOA-K-means averaged only 80% in diagonal accuracy, and recognition accuracy for the fifth feature was just 74%, showing poor robustness. In contrast, Fig 8(d) showed that Fast K-means++ had nearly blank non-diagonal areas (misclassification rate <5%) and an average diagonal accuracy of 97%, clearly surpassing the others. These results showed that Fast K-means++ achieved high robustness and precision in feature recognition.

### 4.2. Application analysis of FK-Stacking enterprise service user intent prediction model

After validating the performance of Fast K-means++, the study further tested the FK-Stacking model by comparing it with models based on Linear Regression (LR), Autoregressive Integrated Moving Average (ARIMA), and Bayesian Optimization (BO). The accuracy, recall, and harmonic mean of precision and recall (F1 Score, F1) of several models in different datasets are shown in Table 2.

**Table 2:** Comparison of predictive performance of different models on various e-commerce datasets.

Dataset	Evaluation metric	FK-Stacking	LR	ARIMA	BO
Alibaba.com	Accuracy	0.98	0.93	0.97	0.95
	Recall	0.97	0.92	0.92	0.94
	F1	0.96	0.9	0.91	0.92
Alimama	Accuracy	0.97	0.91	0.95	0.94
	Recall	0.96	0.90	0.91	0.93
	F1	0.95	0.89	0.90	0.91

*Note: Alibaba.com can be visited from* *https://www.alibaba.com/. The collection and analysis method of the dataset in this research all complied with the terms and conditions for the source of the data.*

**Fig 9:**
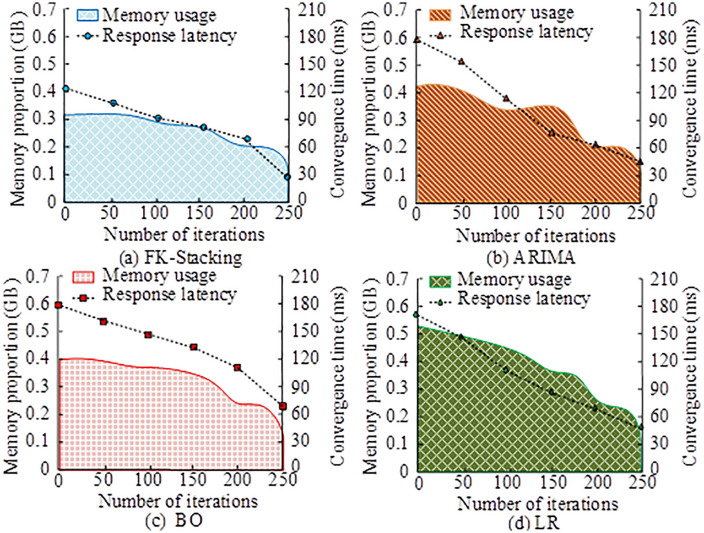
Changes in response time and memory usage with increasing iterations.

According to Table 2, the FK Stacking model achieved optimal performance on all datasets and evaluation metrics, demonstrating excellent and stable predictive ability. On the Alibaba.com dataset, FK Marking has the highest accuracy (0.98) and recall (0.97), reflecting the excellent balance of the model in accurately identifying user intent and comprehensively capturing positive samples. Especially in terms of recall rate, FK Stacking leads the second best performing BO model by 0.03. On the Alimama dataset, the advantage of FK Stacking continues, with an accuracy (0.97) and recall (0.96) that are equally ahead of other models. It is worth noting that the linear regression (LR) model performs the worst in all cases, highlighting the limitations of traditional linear models and the necessity of adopting advanced methods such as ensemble learning when facing complex user behavior data. In summary, the research model can more effectively learn patterns from e-commerce user data, and its predictive performance is significantly and stably superior to traditional and single optimization models, providing a reliable technical solution for precision marketing and service optimization of enterprises. Given the computational efficiency of Fast K-means++, the study also tested model lightweight performance. Changes in response time and memory usage across iterations are shown in Fig 9.

**Fig 10:**
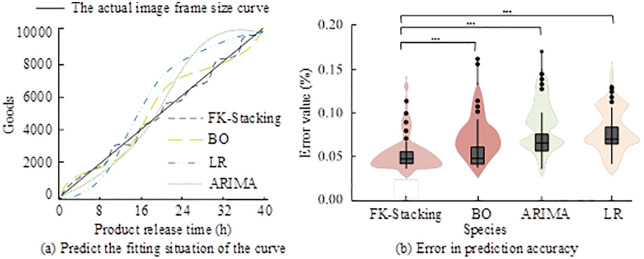
Prediction accuracy error and purchase intent fitting trajectory.

As shown in Fig 9(a), the memory usage of FK-Stacking continuously decreased with more iterations, along with the response time. It started at 0.32 GB and dropped to 0.16 GB after 200 iterations, with the response time reduced to 30.3 ms. In Fig 9(b), ARIMA began with a response time of 172.5 ms and later stabilized at 45.7 ms. In Fig 9(c), BO started with 0.40 GB memory, 0.08 GB higher than FK-Stacking, and dropped to 0.26 GB after optimization, showing less efficient lightweight performance. In Fig 9(d), LR had the highest initial response time and memory usage. After optimization, the response time dropped significantly to 53.2 ms, but memory usage remained high. These findings showed that FK-Stacking achieved efficient model lightweighting through iterative optimization, significantly reducing both memory usage and response latency, validating the performance gains from Fast K-means++. The study also compared the prediction curve of user purchase intent over time and the real purchase counts, as well as the accuracy errors among models. The results are presented in Fig 10.

As shown in Fig 10(a), at the 8-hour mark, FK-Stacking predicted 1950 purchases compared to the actual value of 2000, achieving a 97.5% fit. BO overestimated by 10.3%, while LR and ARIMA predicted only 1803 and 1837, respectively. At the 40-hour mark, FK-Stacking estimated 9803 purchases, with only a 2.1% error, showing the best fit across the entire time range. Fig 10(b) showed that FK-Stacking had a median prediction error of 0.05%, with an interquartile range of 0.03%, minimal dispersion, and no outliers. In contrast, LR had a median error of 0.10% with large dispersion and scattered error distribution. These results indicated that FK-Stacking achieved superior fitting performance and error control, offering a more accurate time-series prediction solution for enterprise user purchase intent.

## 5. Conclusions

To improve the efficiency and accuracy of enterprise user intent prediction, this study proposed a FK-Stacking model that integrated Fast K-means++ with Stacking ensemble learning. Fast K-means++ enhanced the efficiency of feature extraction from large-scale enterprise service data. Meanwhile, the model combined XGBoost and other base classifiers to construct a Stacking-based weighted ensemble learner, which strengthened the prediction capability after multidimensional feature fusion. The experimental results showed that FK-Stacking achieved an accuracy of 0.98, a recall of 0.97, and an F1-score of 0.97 on the Alibaba.com dataset, with improvements of 5%, 5.2%, and 5.1% respectively compared to the Linear Regression model. In terms of time-series fitting, FK-Stacking maintained a prediction deviation of less than 2.1% within 40 hours after product release. The median prediction error was 0.05%, and the interquartile range was 0.03%, showing significantly better stability than other models. In the lightweight test, the memory usage dropped from 0.32 GB to 0.16 GB after 200 iterations, and the response time decreased to 30.3 ms, resulting in more than 60% improvement in computational efficiency. These results demonstrated that the proposed model offered lightweight and high-accuracy advantages in enterprise user data analysis and intent prediction. Although FK Stacking performs well on standard datasets, there is still room for improvement in its clustering performance on non convex data. In the future, feature extraction modules based on deep learning or dynamic weighting strategies for optimizing grid partitioning can be introduced to further enhance the model’s adaptability to complex data structures and improve generalization performance. Specifically, explore the embedding of deep learning feature extraction modules to better capture non-linear high-order features of user behavior; Develop a more flexible dynamic grid partitioning mechanism to meet the clustering needs of non convex and imbalanced data; Expand the model’s ability to fuse multimodal data and achieve joint modeling of text, sequence, and graph structured data; Research real-time and incremental learning mechanisms to enable models to adapt to the dynamic evolution of user behavior.
